# Implementation of an Enhanced Recovery After Surgery Pathway for Pediatric Surgical Oncology using Quality Improvement Methodology

**DOI:** 10.1245/s10434-025-18403-5

**Published:** 2025-10-08

**Authors:** Shachi Srivatsa, Jennifer H. Aldrink, Elizabeth Schneider, Taha Akbar, Giorgio Veneziano, Vanessa Ng, Cindy McManaway, Dana Schwartz, Kris Jatana, Sara A. Mansfield

**Affiliations:** 1 Center for Surgical Outcomes Research, Abigail Wexner Research Institute, Nationwide Children’s Hospital, Columbus, OH USA; 2 Division of Pediatric Surgery, Department of Surgery, Nationwide Children’s Hospital, The Ohio State University College of Medicine, Columbus, OH USA; 3 Center for Clinical Excellence, Nationwide Children’s Hospital, Columbus, OH USA; 4 Department of Anesthesiology and Pain Medicine, Nationwide Children’s Hospital, The Ohio State University College of Medicine, Columbus, OH USA; 5 Division of Pediatric Surgery, Department of Surgery, Nationwide Childrens Hospital, Columbus, OH USA; 6 Department of Otolaryngology, Nationwide Children’s Hospital, The Ohio State University College of Medicine, Columbus, OH USA

**Keywords:** ERAS, Pediatric surgical oncology, Regional anesthesia

## Abstract

**Background:**

Enhanced recovery after surgery (ERAS) pathways have demonstrated significant benefits, but often face challenges in implementation due to the scope of process measures and multidisciplinary buy-in required. This study aimed to standardize surgical care for children undergoing solid tumor resection by implementing an ERAS for Tumor (ERAST) pathway.

**Patients and Methods:**

Our ERAST pathway consisted of 20 process measures. Plan-Do-Study-Act (PDSA) cycles were utilized, including implementing standardized pre- and postoperative orders, data-enabled electronic progress note templates, and multidisciplinary preoperative team huddles. Our primary outcome was 80% adherence to protocol process measures. Secondary outcomes included hospital length-of-stay (LOS) and opioid usage. Balancing measures included readmission and/or emergency room visits within 30 days post-procedure.

**Results:**

Over 15 months, 57 patients (63 surgeries) were included. Median adherence to process measures was 89.5%. Intraoperative fluid administration decreased from 12.19 to 5.97 ml/kg/h (*p* < 0.001). Intraoperative opioid use in abdominal cases fell from 0.37 to 0.24 OME/kg (*p* = 0.0008); postoperative opioid use dropped from 0.16 to 0.04 OME/kg/day (*p* < 0.001). Thoracic cases saw post-operative opioid use decrease from 0.30 to 0.13 OME/kg/day (*p* = 0.0017). Median LOS decreased for laparotomy (4.48–2.87 days), thoracotomy (3.37–2.26 days), and thoracoscopy (1.60–1.15 days), all *p* < 0.001. There was no difference in readmission and/or emergency room visits pre/post ERAST for all cases.

**Conclusions:**

The ERAST pathway achieved high protocol adherence and led to significant reductions in opioid use and LOS, without worsening balancing measures. This demonstrates the effectiveness of multidisciplinary, protocol-driven recovery pathways in pediatric surgical oncology.

**Supplementary Information:**

The online version contains supplementary material available at 10.1245/s10434-025-18403-5.

Enhanced recovery after surgery (ERAS) protocols are evidence-based, multimodal care pathways designed to optimize perioperative outcomes by reducing surgical stress and promoting faster recovery. Initially developed for adult surgical populations, ERAS protocols have demonstrated significant improvements in clinical outcomes, including reduced length of hospital stay (LOS), decreased postoperative complications, and minimized opioid use.^1,2^ The success of ERAS in adult surgical care is attributed to its focus on standardizing perioperative practices through multidisciplinary coordination and adherence to best-practice guidelines.^3,4^ Despite these benefits, the application of ERAS principles in pediatric surgical populations, particularly in oncologic resections, remains limited owing to unique challenges such as variability in patient size, developmental differences, and the complex, multidisciplinary nature of pediatric surgical oncology care.^5–8^

Studies evaluating pediatric ERAS protocols have largely focused on gastrointestinal surgeries, Nuss procedures or pilot programs, leaving a critical need for comprehensive, tumor-specific protocols in pediatric populations.^9–19^ Furthermore, barriers to implementation, including limited provider familiarity, logistical constraints, and inconsistent multidisciplinary buy-in, have hindered broader adoption.^7,11^ This lack of standardization results in significant variability in perioperative care, leading to inconsistent clinical outcomes. A recent multi-institutional study by Mansfield et al. highlighted essential insights into the implementation of ERAS protocols for pediatric patients with abdominal solid tumors.^5,6^ Focusing on abdominal tumor resections, this study demonstrated that a standardized ERAS protocol can significantly reduce complications, opioid consumption, and time to mobility and diet resumption. These findings emphasize the feasibility and effectiveness of ERAS implementation in pediatric surgical oncology, providing a structured approach to improving clinical outcomes.

The objective of this study was to design, implement, and refine an enhanced recovery after surgery for tumors (ERAST) pathway specifically tailored for pediatric patients undergoing abdominal or thoracic tumor resections at a tertiary care children’s hospital. Using a quality improvement (QI) framework, the team aimed to standardize surgical care by achieving at least 80% adherence to 20 defined process measures. Secondary goals included reducing LOS and perioperative opioid use while maintaining or improving balancing measures such as readmission and emergency department (ED) visit rates. This initiative reflects a novel application of QI principles to the development and implementation of a pediatric-specific ERAS protocol, addressing key challenges in pediatric surgical oncology care and paving the way for broader protocol adoption in similar clinical settings.

## Patients and Methods

This study was conducted at a tertiary children’s hospital. Institutional Review Board approval was obtained, and the project was classified as a nonresearch QI initiative, exempt from formal human subject research oversight. A multi-disciplinary team was created with representation from surgical oncology, medical oncology, anesthesiology, acute pain management team, clinic nursing, preoperative nursing, postoperative floor nursing, and inpatient advanced practice providers. Baseline data were obtained by retrospective chart review of all patients undergoing abdominal or thoracic tumor resections between 2015 and 2022. Data obtained included surgical details, intraoperative pain medication usage, intraoperative fluid administration, time to diet, fluid balance, usage of perioperative multimodal pain regimen, and the balancing measures of 30-day readmission, ED visits, and complication rates.

**Fig. 1:**
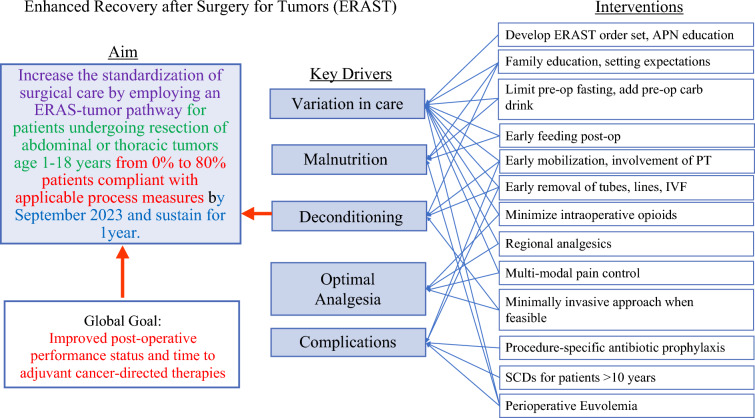
ERAST key driver diagram

A previously published protocol was adapted to meet our institution-specific needs.^5,6^ We also incorporated results from a modified Delphi process review of pediatric ERAS undertaken within the American Pediatric Surgical Association.^7^ Key drivers were identified (Fig. 1). Interventions targeted 20 process measures spanning pre-, intra-, and postoperative phases (Table 1). Preoperative measures included patient and family counseling, carbohydrate-loading up to 2 h before surgery, avoidance of prolonged fasting, and appropriate antibiotic prophylaxis. Preoperative antibiotic prophylaxis was administered according to CDC guidelines for surgical site infection prevention;^20^ for patients with specific clinical concerns, antibiotic selection was tailored based on culture results or empiric therapy as indicated. Intraoperative measures focused on the use of regional anesthesia, use of minimally invasive surgical approach when feasible, maintenance of normothermia, judicious fluid management, and minimization of opioid use through multimodal analgesia. Although the choice of minimally invasive surgery was primarily based on oncologic and anatomic considerations, MIS was included as an ERAST process measure to encourage its use when clinically feasible, given its association with reduced surgical stress, postoperative pain, and length of hospital stay, consistent with ERAS principles. Postoperative measures emphasized early enteral/oral feeding, early mobilization with physical therapy consultation, multimodal analgesia, and prompt removal of intravenous lines, drains, and catheters as clinically appropriate.

**Table 1:** ERAST protocol and definitions for each process measure

ERAST process measure	Minimum required to meet goal
1. Preoperative counseling	Provide ERAST brochure, discuss expectations
2. Carbohydrate load	For patients >6 months encourage carbohydrate-rich, clear liquid the morning of surgery up to 2 hours prior to case start
3. Avoid prolonged fasting	Regular diet night before, no prolonged clear-liquid diet Inpatient orders to allow clears until 2 hours pre-op
4. No bowel prep	Should be avoided unless colon resection planned
5. Antibiotic prophylaxis	Case-appropriate within 60 min prior to incision
6. Regional anesthesia	Pre-incision epidural for open cases, intercostal nerve cryoablation for thoracotomies If contraindication, consider wound catheters, pre-incision TAP or QL blocks +/- TAP/QL/ESP catheters
7. Euvolemia	IVF 3–7 mL/kg/hr (over total OR time)
8. Normothermia	Patient temp 36–38 °C (over total OR time)
9. Minimizing intraoperative opioids	< 0.3 mg/kg IV morphine equivalents
10. Minimally invasive approach	Laparoscopic or robot-assisted when oncologically feasible
11. DVT prophylaxis	SCDs on all patients age ≥ 10 years Pharmacologic prophylaxis: optional at clinician discretion
12. PONV prophylaxis	Emend, Dexamethasone, or Zofran given pre- or intra-operatively
13. Avoid nasogastric tube	May use NG/OG during case, remove prior to leaving OR
14. Postoperative nausea prophylaxis	Prn ondansetron, Benadryl, etc. ordered for ongoing nausea control
15. Early feeding	clears on POD#0, Regular on POD#1. May order sooner as patient tolerates.
16. Early mobilization	Out of bed by POD#1 (document) Physical therapy consult
17. Early removal of IVF	Goal to saline lock on POD#1-2 unless clinically indicated
18. Early removal of drains	Early removal of foley (POD#0-1) based on epidural level (most are thoracic), if unsure ask pain team, Chest tubes removed POD1 if no air leak and volume low
19. Non-opioid pain meds	Scheduled acetaminophen and ketorolac upon leaving the OR unless contraindication. Change to PRN based on need.
20. Minimize opioids	Keep < 0.15 mg/kg IV morphine milligram equivalents per day

*TAP* transversus abdominus plane; *QL* quadratus lumborum; *ESP* erector spinae plane; *PONV* postoperative nausea and vomiting; *IVF* intravenous fluid; *DVT* deep venous thrombosis; *SCD* sequential compression device; *NG* nasogastric; *OG* orogastric; *POD* postoperative day

An impact-effort matrix was utilized to guide implementation. A standardized order set was created within the electronic medical record (EMR). This was high impact for standardization of care, but with moderate effort upfront in collaboration with our EMR build team. During review of baseline data, we noted that several variables of interest were infrequently recorded. Therefore, we created a data-enable progress note template to capture variables such as time to ambulation, diet advancement and tolerance details, and pain control details. An automated report was then generated from these notes to track protocol adherence. These changes sought to minimize manual chart reviews and allow for efficient real-time monitoring.

The prospective study included pediatric patients aged 1 to 18 years undergoing elective abdominal or thoracic tumor resections from September 2023 to December 2024. Patients were excluded if they underwent emergent surgeries. The primary outcome was adherence to the defined process measures, with a target of achieving at least 80% adherence per patient. Specific definitions for each process measure are presented in Table 1. Secondary outcomes included LOS and perioperative opioid use, measured in morphine milligram equivalents per kilogram (MME/kg). Balancing measures included readmissions and emergency department visits within 30 days postoperatively. A report card was created to track monthly adherence to all process measures.

This study employed a quality improvement framework using the Plan-Do-Study-Act (PDSA) cycles, a well-established iterative framework for testing and refining changes in healthcare systems.^21^ The PDSA approach involves four steps: planning an intervention on the basis of identified problems, executing it on a small scale, studying the outcomes and data, and then acting to refine, expand, or abandon the change based on the results. Early in the prospective cohort it was noted that several intraoperative goals were not being routinely met. Therefore, a preoperative huddle with operating room (OR) staff, anesthesia team, and surgeon was initiated as the first PDSA cycle. This huddle included discussion of optimizing operating room ambient temperature to achieve consistent patient normothermia, goals for weight-based fluid and opioid administration, need for sequential compression devices, and a regional analgesia plan. A huddle rubric was created that automatically calculated weight-based goals. The second cycle implemented an EMR-based progress note template and automated reports, improving the consistency of postoperative documentation and reducing the need for manual chart review. These iterative cycles allowed the ERAST protocol to evolve in response to practical challenges encountered during implementation.

Charts were reviewed for postoperative complications within 30-days of surgery on the basis of previously published list of Clavien–Dindo classification. Readmissions for chemotherapy or chemotherapy related issues were not included. Data were collected prospectively from EMRs using a standardized data collection template. Descriptive statistics summarized baseline patient characteristics and adherence rates, while continuous variables were analyzed using paired *t*-tests or Wilcoxon rank-sum tests where appropriate. Results are reported on the basis of SQUIRE 2.0 reporting standards for QI projects.^22^

## Results

### Baseline Characteristics

**Fig. 2:**
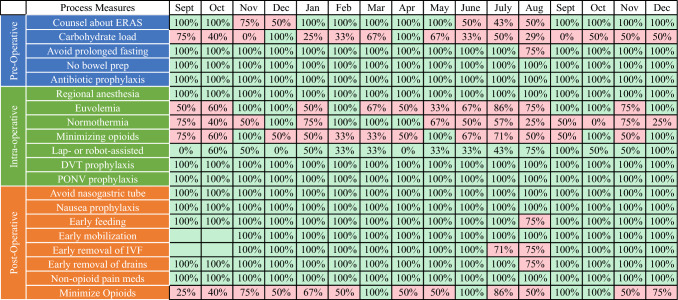
Report card of adherence to process measures through the intervention period

The baseline cohort included 282 patients who underwent a total of 353 unique thoracic or abdominal tumor resections. The prospective cohort included 57 patients who underwent 63 unique thoracic or abdominal tumor resections. The median adherence in the prospective cohort to the 20 defined process measures was 89.5% (interquartile range (IQR) 85.0–94.4%), surpassing the target of 80% adherence. The report card demonstrates adherence to process measures across the intervention period (Fig. 2).

The procedures in the baseline cohort comprised 79 thoracoscopies, 93 thoracotomies, 39 laparoscopies, and 142 laparotomies. The prospective cohort’s procedures included 13 thoracoscopies, 12 thoracotomies, 10 laparoscopies, and 28 laparotomies. Both cohorts included a range of complex surgical cases, such as thoracoscopic and open thoracotomy for mass removal and metastasectomies, as well as laparoscopic and open laparotomy procedures for abdominal and pelvic tumors (Table 2). The pathologic diagnoses and distributions in each cohort are presented in Supplementary Table 1.

**Table 2:** Surgical procedures performed for baseline and ERAST cohorts

	Baseline (*N*, %)	ERAST (*N*, %)
*Thoracoscopy*		
Wedge resection	63 (79.7%)	10 (76.9%)
Mediastinal/paraspinal mass excision	14 (17.7%)	3 (23.1%)
Pneumonectomy	2 (2.5%)	0 (0.0%)
*Thoracotomy*		
Wedge resection	70 (75.3%)	10 (83.3%)
Mediastinal mass resection	9 (9.7%)	0 (0.0%)
Lobectomy	9 (9.7%)	2 (16.7%)
Mass excision and chest wall reconstruction	5 (5.4%)	0 (0.0%)
*Laparoscopy*		
Mass removal	15 (38.5%)	3 (30.0%)
Adrenalectomy	14 (35.9%)	2 (20.0%)
Unilateral or bilateral oophorectomy and/or salpingectomy	7 (17.9%)	5 (50.0%)
Appendectomy	1 (2.6%)	0
Colectomy	2 (5.1%)	0
*Laparotomy*		
Mass excision (including neuroblastoma/retroperitoneal masses)	46 (32.4%)	6 (21.4%)
Radical nephrectomy or nephrectomy with ureterectomy	45 (31.7%)	11 (39.3%)
Partial nephrectomy	12 (8.5%)	2 (7.1%)
Partial hepatectomy	9 (6.3%)	0 (0.0%)
Ovarian cystectomy	8 (5.6%)	5 (17.9%)
Adrenalectomy	7 (4.9%)	0 (0.0%)
Unilateral or bilateral oophorectomy and/or salpingectomy	6 (4.2%)	3 (10.7%)
Small intestine excision with anastomosis	5 (3.5%)	0 (0.0%)
Colectomy	2 (1.4%)	1 (3.6%)
Splenectomy	1 (0.7%)	0 (0.0%)
Cystectomy with neobladder creation	1 (0.7%)	0 (0.0%)

### Fluid Administration

Significant reductions in intraoperative fluid administration were observed across all surgical groups (Table 3). Postoperative fluid balance was recorded as the total fluid balance for the hospital stay up to postoperative day three.

**Table 3:** Comparison of key process and balancing measures between the pre and post intervention cohorts

		Thoracoscopy Baseline: *N* = 79 ERAST: *N* = 13 (Median, IQR) or (*N*, %)	*p*-value	Thoracotomy Baseline: *N* = 93 ERAST: *N* = 12 (Median, IQR) or (*N*, %)	*p*-value
Intraoperative fluid (ml/kg/h)	Baseline ERAST	12.67 (7.82, 20.20) 6.15 (3.89, 8.05)	< 0.001	9.10 (5.01, 12.34) 5.36 (3.94, 5.54)	0.016
Intraoperative opioid (OME/kg)	Baseline ERAST	0.31 (0.17, 0.46) 0.30 (0.28, 0.38)	0.865	0.33 (0.24, 0.47) 0.24 (0.18, 0.35)	0.158
Postoperative opioid (OME/kg/day)	Baseline ERAST	0.36 (0.16, 0.57) 0.08 (0.05, 0.13)	< 0.001	0.24 (0.11, 0.43) 0.16 (0.11, 0.34)	0.333
Time to regular diet (hours:minutes)	Baseline ERAST	2:37 (0:23 5:58 4:45 (2:21, 7:39)	0.412	5:03 (1:42, 15:39) 5:49 (3:47, 10:08)	0.562
POD 0 fluid balance (mL)	Baseline ERAST	913 (573, 1397) 818 (686, 1352)	0.810	1342 (695, 1771) 1385 (963, 2122)	0.347
POD 1 fluid balance (mL)	Baseline ERAST	289 (-86, 640) -101 (-328, 345)	0.032	648 (60, 1057) 445 (186, 1258)	0.697
POD 2 fluid balance (mL)	Baseline ERAST	NA		- 36 (-555, 270) -180 (-220, 150)	0.826
LOS (days)	Baseline ERAST	1.60 (1.37, 2.48) 1.15 (0.99, 1.40)	< 0.001	3.37 (2.56, 4.51) 2.26 (1.75, 2.83)	< 0.001
All-cause 30-day readmission/ED visit	Baseline ERAST	5 (6.4%) 3 (23.1%)	0.084	16 (17.2%) 0 (0%)	0.206
Surgery related 30-day readmission/ED visit	Baseline ERAST	3 (3.8%) 3 (23.1%)	0.464	14 (15.1%) 0 (0.0%)	1.00
All-cause 30-day complication	Baseline ERAST	10 (12.8%) 2 (15.4%)	0.680	16 (17.2%) 1 (8.3%)	0.686

		Laparoscopy Baseline: *N* = 39 ERAST: *N* = 10 (Median, IQR) or (*N*, %)		Laparotomy Baseline: *N* =142 ERAST: *N* = 28 (Median, IQR) or (*N*, %)	
Intraoperative fluid (ml/kg/h)	Baseline ERAST	13.38 (7.07, 18.61) 3.87 (3.09, 5.45)	< 0.001	14.96 (9.06, 21.56) 6.65 (4.62, 9.20)	< 0.001
Intraoperative opioid (OME/kg)	Baseline ERAST	0.32 (0.22, 0.42) 0.22 (0.11, 0.26)	0.025	0.38 (0.24, 0.59) 0.25 (0.18, 0.39)	0.007
Postoperative opioid (OME/kg/day)	Baseline ERAST	0.07 (0.03, 0.27) 0.02 (0.00, 0.04)	0.028	0.18 (0.07, 0.40) 0.05 (0.03, 0.09)	< 0.001
Time to regular diet (hours:minutes)	Baseline ERAST	3:39 (1:03, 15:23) 2:50 (1:50, 3:28)	0.617	15:32 (2:47, 17:25) 10:20 (1:53, 19:36)	< 0.001
POD 0 fluid balance (mL)	Baseline ERAST	923 (516, 1738) 1320 (873, 2088)	0.472	860 (429, 1525) 834 (452, 1089)	0.490
POD 1 fluid balance (mL)	Baseline ERAST	154 (-207, 442) -65 (-932, 595)	0.459	526 (57, 1066) 602 (56, 1238)	0.912
POD 2 fluid balance (mL)	Baseline ERAST	NA		-61 (349, 327) -107 (512, 342)	0.772
POD 3 fluid balance (mL)	Baseline ERAST	NA		-104 (455, 195) -385 (-702, -164)	0.056
All-cause 30-day readmission/ED visit	Baseline ERAST	5 (12.8%) 3 (30.0%)	0.333	16 (11.3%) 3 (10.7%)	1.00
Surgery related 30-day readmission/ED visit	Baseline ERAST	2 (5.1%) 1 (10.0%)	1.00	13 (9.2%) 1 (3.6%)	0.061
30-day complication	Baseline ERAST	6 (15.4%) 0 (0%)	0.324	61 (43.0%) 8 (28.6%)	0.207

**Thoracoscopy**: median intraoperative fluid administration decreased from 12.67 ml/kg/h (IQR 7.82–20.20) in the baseline cohort to 6.15 ml/kg/h (IQR 3.89–8.05) with ERAST in thoracoscopy cases (*p* < 0.001). There was no significant difference in median post-operative fluid balance on POD 0 for thoracoscopy cases (*p* = 0.81). There was a significant difference in median postoperative fluid balance on POD 1 for thoracoscopy cases with a median of 289 mL in the baseline cohort and −101mL in the ERAST cohort (*p* = 0.032).

**Thoracotomy**: similarly, thoracotomy cases saw a reduction in intraoperative fluid administration from 9.10 ml/kg/h (IQR 5.01–12.34) to 5.36 ml/kg/hr (IQR 3.94–5.54; *p* = 0.016). There was no significant difference in either POD 0 or POD 1 fluid balances in the thoracotomy cases (both *p* > 0.05).

**Laparoscopy**: in laparoscopic cases, intraoperative fluid administration decreased from 13.38 ml/kg/h (IQR 7.07–18.61) at baseline to 3.87 ml/kg/h (IQR 3.09–5.45) with ERAST. There were no significant differences in the POD 0 or POD 1 fluid balances in the laparoscopy cases (both *p* > 0.05).

**Laparotomy**: in laparotomy cases, intraoperative fluid administration decreased from 14.96 ml/kg/h (IQR 9.06–21.56) at baseline to 6.65 ml/kg/h (IQR 4.62–9.20). There were no significant differences in the POD 0–POD 3 fluid balances in the laparotomy cases (all *p* > 0.05).

### Analgesia Approaches and Opioid Utilization

In the ERAST cohort, 100% of patients received either regional or local analgesia as part of their perioperative pain regimen. When considering all open cases, regional anesthetic methods—including regional anesthesia catheters and cryoablation—were used in 90.0% (*n* = 195) of cases in the baseline cohort, which was not significantly different from the ERAST cohort’s usage of 100% (*n* = 40). Postoperative opioid use significantly declined in both thoracic and abdominal tumor resections (Table 3). Ketorolac was administered in 42.5% (*n* = 150) of the baseline cohort cases which was significantly less than the 93.7% (*n* = 59) administered in the ERAST cohort (*p* < 0.001). Acetaminophen was administered in 96.9% (*n* = 342) of the baseline cohort cases which was similar to the 98.4% (*n* = 62) in the ERAST cohort (*p* = 1.00). Gabapentin was utilized in 61.9% (*n* = 39) of ERAST cases, but this data were not collected in the baseline group for comparison.

**Thoracoscopy**: among thoracoscopy cases, none received regional anesthesia, but all received local anesthetic administration. This was not significantly different from the baseline cohort, where 3.8% (*n* = 3) received regional anesthesia (*p* = 1.00). Intraoperative opioid administration did not differ significantly (*p* = 0.865). Postoperative opioid use decreased from 0.36 OME/kg/day (IQR 0.16–0.57) to 0.08 OME/kg/day (IQR 0.05–0.13; *p* < 0.001).

**Thoracotomy**: for thoracotomy cases in the ERAST cohort, 66.7% (*n* = 8) received intercostal nerve cryotherapy with intercostal nerve blocks, while 33.3% (*n* = 4) received an epidural catheter. One patient received dual regional anesthesia with both cryotherapy and a peripheral nerve catheter. In the baseline cohort, 88.2% (*n* = 82) received regional anesthesia. The overall use of regional anesthesia was significantly different between the baseline and ERAST thoracotomy cohorts, with rates of 88.2% (*n* = 82) and 91.7% (*n* = 11), respectively (*p* < 0.001). This is likely explained by the introduction and increasing utilization of cryotherapy in the ERAST cohort. Intraoperative opioid administration did not differ significantly (*p* = 0.158). There was no significant difference in overall post-operative opioid use in thoracotomy cases (*p* = 0.333). When comparing patients who received cryotherapy to those who did not, no significant difference in postoperative opioid use was found between the baseline cohort and the ERAST cohort with cryotherapy (*p* = 0.066) or between the baseline cohort and the ERAST cohort without cryotherapy (*p* = 0.617).

**Laparoscopy**: similarly, in laparoscopy cases, all ERAST cohort patients received local anesthetic, while none received regional anesthesia, which was comparable to the baseline cohort’s 2.6% (*n* = 1) usage (*p* = 1.00). Intraoperative opioid use decreased from a median of 0.32 OME/kg (IQR 0.22–0.42) baseline to 0.22 OME/kg (IQR 0.11–0.26) ERAST (*p* = 0.025). Median postoperative opioid use decreased from 0.07 OME/kg/day (IQR 0.03–0.27) to 0.02 OME/kg/day (IQR 0.00–0.04; *p* = 0.028) in laparoscopy cases.

**Laparotomy**: for laparotomy cases, epidural anesthesia was used in 85.2% (*n* = 22) of ERAST cohort patients, which was similar to the baseline cohort’s usage of 80.0% (*n* = 113) (*p* = 1.00). The median intraoperative opioid administration decreased from 0.38 OME/kg (IQR 0.24–0.59) to 0.25 OME/kg (IQR 0.18–0.39; *p* = 0.007). Postoperative opioid administration decreased from a median of 0.18 OME/kg/day (IQR 0.07–0.40) to 0.05 OME/kg/day (IQR 0.03–0.09; *p* < 0.001).

### Diet Advancement

There was no significant difference in time to placement of regular diet order between baseline and ERAST patients for those undergoing thoracoscopy, thoracotomy, or laparoscopy (all *p* > 0.05) (Table 3). There was a significant reduction in time to regular diet for those undergoing laparotomy ERAST (median 10 h 20 min from OR) compared with baseline (median 15 h and 32 min from OR) (*p* < 0.001). There were no significant differences in rates of postoperative nausea/vomiting (PONV) between any baseline and ERAST cohort.

### Other Process Measures

For the following process measures, there were no comparative measures in the baseline cohort. Eighty one percent of cases received counseling on the ERAST pathway prior to day of surgery. All patients in the ERAST cohort were encouraged to consume a carbohydrate load the morning of surgery, and 46% followed the instruction. No patient in the ERAST pathway underwent bowel preparation. All patients in the ERAST cohort received appropriate antibiotic prophylaxis. All patients >10 years of age received venous thromboembolism prophylaxis with sequential compression devices intra-operatively. All patients received administration of PONV prophylaxis in the OR and postoperatively. All patients were mobilized out of bed by post-operative day (POD) 1. All patients undergoing thoracoscopy, thoracotomy or laparoscopy had discontinuation of their intravenous fluids by POD 1. For those undergoing laparotomy, 93% had discontinuation of their intravenous fluids by POD 2, with 100% by POD 3.

### Complications

There were no significant differences in 30-day readmission/ED visit or overall complication rates following ERAST implementation (Table 4).

**Table 4:** Comparison of complications between baseline and ERAST cohorts

Complication type		Thoracoscopy Baseline: *N* = 79 ERAST: *N* = 13 (*n*, %)	*p*-value	Thoracotomy Baseline: *N* = 93 ERAST: *N* = 12 (*n*, %)	*p*-value
Grade 1	Baseline ERAST	8 (10.1%) 1 (7.7%)	1.00	8 (8.6%) 1 (8.3%)	1.00
Grade 2	Baseline ERAST	1 (1.3%) 0 (0.0%)	1.00	2 (2.2%) 0 (0.0%)	1.00
Grade 3	Baseline ERAST	0 (0.0%) 0 (0.0%)	1.00	2 (2.2%) 0 (0.0%)	1.00
Grade 4	Baseline ERAST	1 (1.3%) 0 (0.0%)	1.00	1 (1.1%) 0 (0.0%)	1.00

		Laparoscopy Baseline: *N* = 39 ERAST: *N* = 10 (*n*, %)		Laparotomy Baseline: *N* = 142 ERAST: *N* = 28 (*n*, %)	
Grade 1	Baseline ERAST	4 (10.3%) 0 (0.0%)	1.00	49 (34.5%) 3 (10.7%)	0.018
Grade 2	Baseline ERAST	0 (0.0%) 0 (0.0%)	1.00	8 (5.6%) 2 (7.1%)	0.327
Grade 3	Baseline ERAST	1 (2.6%) 0 (0.0%)	1.00	2 (1.4%) 0 (0.0%)	1.00
Grade 4	Baseline ERAST	1 (2.6%) 0 (0.0%)	1.00	2 (1.4%) 3 (10.7%)	< 0.001

Of those who returned to the ED or required readmission, surgery related events accounted for 60.0% (*n* = 3/5) of the readmission rate at baseline and 100.0% (*n* = 3/3) ERAST in thoracoscopy cases (*p* = 0.464). In thoracotomy cases, surgery related readmission/ED visits decreased from 87.5% (*n* =14/16) of the readmission rate to 0% (*n* = 0/0) (*p* = 1.00). In laparoscopy cases, surgery related readmission/ED visits were 40.0% (*n* = 2/5) of the readmission rate at baseline and 33.3% (n=1/3) ERAST (*p* = 1.00). In laparotomy cases, surgery related readmission/ED visits were 81.3% (*n* = 13/16) of the readmission rate at baseline and 33/3% (*n* = 1/3) ERAST (*p* = 0.061). Examples of specific complications observed in each cohort are presented in Supplemental Table 2.

### Length of Stay

Significant reductions in hospital LOS were achieved with the ERAST pathway. Thoracoscopy cases experienced a reduction of median LOS from 1.60 days (IQR 1.37–2.48) to 1.15 days (IQR 0.99–1.40; *p* < 0.001). Similarly, LOS after thoracotomy decreased from 3.37 days (IQR 2.56–4.51) to 2.26 days (IQR 1.75–2.83; *p* < 0.001). Median LOS for laparoscopy cases did not decrease significantly from baseline (1.28 days, IQR: 1.09–1.76) to ERAST (0.97 days, IQR: 0.70–1.47) (*p* = 0.162). Median LOS for laparotomy cases decreased from 4.48 days (IQR 3.49–6.23) baseline to 2.87 days (IQR 2.00–3.20; *p* < 0.001).

### Plan-Do-Study-Act Cycles (Table 5)

**Table 5:** PDSA cycles during implementation of the ERAST pathway

PDSA cycle	Intervention
Cycle 1: November 2023	Introduction of the pre-operative huddle to be completed by anesthesia, nursing and surgical staff prior to case start Pre-operative Huddle Rubric: • Patient information: Name, Age, Weight • ERAST Components: • Maintain patient temperature 36-38 degrees Celsius • Is patient greater than or equal to age 10? If yes, place SCDs prior to induction • Fluid goal 3-7ml/kg/hr. Blood loss may be replaced 1:1 with project of choice as needed. Fluid goal provided per patient specifications • Opioid-sparing anesthetic goal: <0.3 mk/kg IV OME. Goals provided by medication per patient specifications • Regional anesthesia plan • Epidural, TAP/ESP/QL catheters before incision. •If contraindications to above – 1-shot block or wound catheters. • If MIS, max dose local anesthetic infiltration of wounds • Adjunct medications, if no contraindications: • Acetaminophen • Ketorolac • Ondansetron – consider additional PONV prophylaxis for older children
Cycle 2: January 2024	Implementation of the ERAST EPIC note to assist with documentation of post-operative measure adherence. APNs and residents on service were educated on usage of the note template The note template was created with input from APNs and nursing staff that routinely care for these patients post-operatively.

A preoperative huddle to improve intraoperative temperature was introduced as part of the first PDSA cycle, conducted by anesthesia, nursing, and surgical teams. Following implementation, adherence to intraoperative normothermia increased.

During the second PDSA cycle, a data-enabled progress note template was implemented within the EMR system to standardize documentation of postoperative process measures. An automated report generated from these notes was sent bi-weekly to track adherence.

## Discussion

Use of a standardized ERAS protocol in pediatric surgical oncology improved length of stay, reduced opioid consumption, improved mobility, and minimized NPO periods without worsening complications or readmissions. Successful implementation of the ERAST pathway was facilitated by QI methodology and comprehensive multidisciplinary engagement from anesthesia, nursing, advanced practice providers, and surgical teams.

The ERAST pathway led to significant improvements across multiple perioperative domains. Adherence to defined process measures surpassed the target, achieving a median adherence of 89.5% (IQR 85.0–94.4%) over the course of the study period. Our findings align with existing research supporting ERAS protocols in surgical populations. Previous studies have demonstrated that ERAS protocols can reduce postoperative complications and enhance recovery in children undergoing various surgical procedures.^5,6,8–10,12–17,23,24^ Our study contributes to this body of evidence by focusing specifically on pediatric patients undergoing abdominal or thoracic tumor resections, a group that has been underrepresented in prior enhanced recovery research. The significant reductions in intraoperative fluid administration and opioid use observed in our study are consistent with findings from other pediatric ERAS studies, which have reported decreased intraoperative fluids and opioid consumption.^6,12^ Furthermore, the decrease in hospital length of stay observed in our study mirrors results from previous research indicating that ERAS protocols can significantly reduce LOS in pediatric surgical patients.^6,8,12^ Our study also addresses the need for procedure-specific ERAS protocols in pediatric surgery, as highlighted in recent literature. By tailoring the ERAS pathway to the unique needs of pediatric patients undergoing tumor resections, we have demonstrated that such customized protocols can lead to improved adherence to process measures and better clinical outcomes.

The ERAST pathway demonstrates the efficacy of a multidisciplinary quality improvement approach in standardizing perioperative care for complex pediatric surgical cases. The use of Plan-Do-Study-Act cycles was instrumental in achieving this success, as it allowed for iterative refinements to interventions based on real-time data and feedback, ensuring adaptability and sustained improvements. The first PDSA cycle introduced preoperative huddles conducted by anesthesia, nursing, and surgical teams. These huddles ensured consistent application of ERAST components. Initially, this intervention significantly improved adherence to intraoperative normothermia, increasing from 0 to 100%. However, adherence subsequently decreased, primarily in cases where patients were transported to the operating room (OR) from interventional radiology (IR) following preoperative tumor localization. This highlighted the need for further interventions tailored to address the challenges in maintaining normothermia during transitions from IR to the OR. In response, a new PDSA cycle is being developed to address temperature management during IR procedures, aiming to improve overall normothermia adherence in these cases. Additionally, we noted that despite patients receiving education on carbohydrate load prior to surgery, many patients did not complete this portion of the pathway. We are developing a new PDSA cycle to assess causes for this and improve adherence.

The second PDSA cycle focused on implementing a data-enabled progress note template within the institution’s EMR system to standardize the documentation of post-operative process measures. Developed collaboratively with advanced practice providers (APPs) and nursing staff, the tool facilitated consistent monitoring of critical practices, such as early mobilization, early removal of intravenous lines, and multimodal pain management. Training sessions were conducted to ensure the effective adoption of the template. An automated report generated by these progress notes was then sent biweekly, limiting the chart review needed to track many of the process measures. A limitation of our baseline data was inconsistent documentation of mobility and tolerance of diet. The exact date that patients were able to get out of bed was rarely documented.

The ERAST pathway facilitated expeditious recovery in children undergoing abdominal or thoracic solid tumor resections, ensuring timely progression to adjuvant treatments, a goal uniquely important to oncology patients. By optimizing intraoperative management and multimodal analgesia, the pathway supported overall stability and wellbeing. Notably, there has been a temporal shift in the last two years towards the use of cryoablation over epidural analgesia for thoracotomy cases, reflecting evolving pain management strategies. The lack of difference in pain control needs with or without cryotherapy may be attributed to the overall impact of the ERAST protocol. These comprehensive improvements in multimodal pain management likely minimized the relative contribution of cryotherapy to postoperative opioid reduction. Early recovery milestones, such as early mobilization and reduced intravenous fluid dependence, contributed to a shorter length of stay without compromising patient safety. These findings underscore the importance of standardized, evidence-based protocols in supporting efficient recovery and continuity of oncologic care.

This study’s strengths include its robust QI framework and the prospective collection of comprehensive process and outcome measures. These elements ensured iterative refinement and consistent monitoring of adherence to the protocol. However, several limitations warrant consideration. First, the single-center design may limit generalizability, particularly to institutions with different resource levels or patient populations. Additionally, while adherence rates were high, certain measures, such as minimizing intraoperative opioid use, exhibited variability across surgical types, underscoring the need for targeted interventions. Lastly, balancing measures, although stable, had small sample sizes in some subgroups, limiting statistical power for detecting differences in complications or readmissions. While no statistically significant differences were observed, the notable discrepancies in percentage changes suggest a potential clinical difference that warrants further monitoring and discussion in future studies.

Future research should focus on exploring strategies to enhance adherence to specific process (such as carbohydrate load) measures to further optimize outcomes. Investigations into long-term outcomes, including functional recovery and quality of life, would also provide valuable insights into the broader impact of ERAS pathways in pediatric oncology. Lastly, integrating advanced digital tools, such as real-time adherence tracking via electronic health record systems, may streamline implementation and improve compliance.

In conclusion, the successful implementation of the ERAST pathway highlights the transformative potential of a multidisciplinary quality improvement approach in optimizing perioperative care for pediatric patients undergoing complex tumor resections. By leveraging quality improvement methodologies and tailoring ERAS principles to the unique needs of this population, the pathway achieved significant improvements in process adherence, perioperative outcomes, and recovery metrics. The ERAST pathway serves as a model for future endeavors aimed at standardizing care, enhancing recovery, and reducing healthcare costs in pediatric oncology and beyond.

## Supplementary Information

Below is the link to the electronic supplementary material.

Supplementary file1 (DOCX 16 KB)
