# Supplementary material for: Implementation of an Enhanced Recovery After Surgery Pathway for Pediatric Surgical Oncology using Quality Improvement Methodology
Source: Ann Surg Oncol. 2025 Oct 8;32(13):10066–76. doi: 10.1245/s10434-025-18403-5 (PMC12589384; doi:10.1245/s10434-025-18403-5)
Supplement: Supplementary file 1 — Supplementary file1 (DOCX 16 KB) [file 10434_2025_18403_MOESM1_ESM.docx]

Supplemental Table 1: Pathologic diagnoses of entire cohort.

| **Pathology** | **Baseline**  **(N, %)** | **ERAST**  **(N, %)** |
| --- | --- | --- |
| Nephroblastoma | 50 (17.7%) | 11 (19.3%) |
| Neuroblastoma | 43 (15.2%) | 3 (5.3%) |
| Ewing sarcoma | 22 (7.8%) | 5 (8.8%) |
| Mature teratoma | 18 (6.4%) | 8 (14.0%) |
| Ganglioneuroblastoma | 16 (5.7%) | 1 (1.8%) |
| Osteosarcoma | 15 (5.3%) | 6 (10.5%) |
| Ganglioneuroma | 6 (2.1%) | 1 (1.8%) |
| Embryonal rhabdomyosarcoma | 5 (1.8%) | 1 (1.8%) |
| Clear cell sarcoma of kidney | 4 (1.4%) | 0 (0%) |
| Hepatoblastoma | 4 (1.4%) | 0 (0%) |
| Mixed germ cell tumor | 4 (1.4%) | 2 (3.5%) |
| Other | 95 (33.7%) | 19 (33.3%) |

Supplemental Table 2: Examples of Complications Observed in Baseline and ERAST Cohorts

| Surgical Group | Cohort | Example Complications Observed |
| --- | --- | --- |
| Thoracoscopy | Baseline | Postoperative fever, pleural effusion |
|  | ERAST | Musculoskeletal chest pain, postoperative fever |
| Thoracotomy | Baseline | Pneumonia, wound infection, prolonged air leak |
|  | ERAST | Musculoskeletal chest pain |
| Laparoscopy | Baseline | Ileus, postoperative bleeding |
|  | ERAST | None reported |
| Laparotomy | Baseline | Fever, wound infection, sepsis, urinary retention |
|  | ERAST | Cardiopulmonary resuscitation, retained wound catheter requiring removal, septic shock |
